# The influence of a ban on outpatient intravenous antibiotic therapy among the secondary and tertiary hospitals in China

**DOI:** 10.1186/s12889-020-09948-z

**Published:** 2020-11-25

**Authors:** Xiaomin Wang, Dan Wu, Ziming Xuan, Weiyi Wang, Xudong Zhou

**Affiliations:** 1 Institute of Social Medicine, School of Medicine, Zhejiang University, 866 Yuhangtang Road, Hangzhou, 310058 China; 2 Department of Clinical Medicine, London School of Hygiene and Tropical Medicine, Keppel Street, London, WC1E 7TH UK; 3 Department of Community Health Sciences, Boston University School of Public Health, 801 Massachusetts Ave, Boston, MA 02118 USA

**Keywords:** China, Antimicrobial stewardship, Intravenous antibiotic use, Antimicrobial resistance

## Abstract

**Background:**

Antimicrobial resistance (AMR) is a serious global public health challenge. Physicians’ over-prescription of antibiotics is a major contributor, and intravenous (IV) antibiotic use has been a particular concern in China. To address the rapid fallout of antibiotic overuse, the Chinese government has piloted a ban of IV antibiotics in the outpatient department (OD) with the exemption of paediatrics, emergency department (ED), and inpatient ward of secondary and tertiary hospitals in several provinces.

**Methods:**

To assess the potential impact of the policy, we conducted a mixed-methods study including 1) interviews about the ban of IV antibiotic use with 68 stakeholders, covering patients, health workers, and policy-makers, from two cities and 2) a hospital case study which collected routine hospital data and survey data with 207 doctors.

**Results:**

Our analyses revealed that the ban of IV antibiotics in the OD led to a reduction in the total and IV antibiotic prescriptions and improved the rational antibiotic prescribing practice in the OD. Nevertheless, the policy has diverted patient flow from OD to ED, inpatient ward, and primary care for IV antibiotic prescriptions. We also found that irrational antibiotic use in paediatrics was neglected. Radical policy implementation, doctors circumvented the regulations, and lack of doctor-patient communication during patient encounters were barriers to the implementation of the ban.

**Conclusions:**

Future efforts may include 1) to de-escalate both oral and IV antibiotic therapy in paediatric and reduce oral antibiotic therapy among adults in outpatient clinics, 2) to reduce unnecessary referrals by OD doctors to ED, primary care, or inpatient services and better coordinate for patients who clinically need IV antibiotics, 3) to incorporate demand-side tailored measures, such as public education campaigns, and 4) to improve doctor-patient communication. Future research is needed to understand how primary care and other community clinics implement the ban.

**Supplementary Information:**

**Supplementary information** accompanies this paper at 10.1186/s12889-020-09948-z.

## Background

Antimicrobial resistance (AMR) is the ability of microorganisms (like bacteria, virus, and some parasites) to respond to the antimicrobial that once could successfully defeat them. AMR resulted in prolonged illness, increased mortality, and higher medical costs, and has become a serious public health challenge worldwide [1, 2]. Previous research showed that an estimate of 10 million people would die each year by 2050 if AMR continues to rise [3]. Annual economic losses due to AMR are 21 to 34 billion US dollars in the United States of America [4] and 1.5 billion euros in Europe [5], respectively. Irrational use of antibiotics accelerates AMR [6]. Drivers for irrational use of antibiotics included physicians’ uncertainty to distinguish bacterial from viral infections, perverse incentives to over-prescribe, patient expectations of antibiotic use, and over-the-counter antibiotic sales in pharmacies [7, 8].

Antimicrobial stewardship programs (ASPs), a systematic approach to rationalize the use of antimicrobial agents [9], are adopted by many countries worldwide to prevent AMR. Evidence has shown that ASPs decreased the quantity and improved the quality of antibiotic prescriptions [10, 11], promoted patient clinical outcomes (e.g., decrease the prevalence of nosocomial infections) [12], and could curtail AMR [13–15]. China has the most rapid growth of AMR in the world [16]. In response to the concern of AMR, the Chinese National Health Commission (NHC, former Ministry of Health) implemented a series of nationwide ASPs from 2004. In 2004, the NHC released a guideline that classified antibiotics into three categories based on the affordability, clinical effectiveness, clinical safety, and AMR concern – non-restricted-, restricted-, and special-grade [17]. Non-restricted antibiotics refer to the antibiotics which are cheap, clinically safe and effective and have little effect on AMR (e.g., penicillin, cephradine, and cefaclor); restricted antibiotics are relatively expensive and with a higher risk of AMR (e.g., mecillinam, ceftazidime, and sultamicillin); special-grade antibiotics are for advanced infections and only recommended for situations when all alternative antibiotics are unlikely to be effective (i.e., meropenem, aztreonam, and faroenem) and can only be prescribed by chief physicians [17, 18]. Two national surveillance systems for clinical antibiotic use [19] and AMR [20] covering 31 provinces and 1412 health facilities were established in 2005. Besides, the NHC initiated zero mark-ups for essential medicines to eliminate the perverse incentives to overprescribe medicines, including antibiotics [21]. In 2011, NHC launched another three-year national scheme to reduce clinical antibiotic use in tertiary hospitals [22–24]. Target antibiotic prescription rates in tertiary hospitals’ outpatient, emergency, and inpatient sections are set at less than 20, 40, and 60% out of the total number of visits in each department respectively. The scheme mainly consists of establishing mandatory administrative strategies for rational antibiotic use, setting targets for antibiotic use management, developing audit and inspection systems, and assigning hospital leadership for achieving these targets.

Overuse of injections when oral formulations would be appropriate is a major form of irrational use of antibiotics [25]. The overuse of intravenous (IV) antibiotics is striking in China [26] where the world’s highest number of total IV antibiotics is consumed each year [27]. The IV antibiotics use rate for upper respiratory tract infections was around 40% in 2010 [21].

In 2016, a few places including Zhejiang, Jiangsu, Shanxi, Anhui, Jiangxi, and Shanghai, piloted a ban on IV antibiotics use for outpatients in secondary and tertiary hospitals (hereafter, the ban) [28]. Before the ban, IV antibiotics could be prescribed by a physician from any outpatient departments and administrated by nurses using an IV drip in hospitals. Since the inception of the ban, physicians in outpatient departments (OD) are not allowed to prescribe IV antibiotics for adult patients with the exception of paediatrics, emergency, and inpatient services [29].

The healthcare system and policy can also affect clinical behaviour*s. China* has a three-tiered healthcare system. Community-based health facilities are expected to provide primary care, while secondary and tertiary hospitals provide referral care. However, there are no effective gate-keeping primary care mechanisms; as a result patients are free to choose whichever health facilities they prefer [30]. Besides, emergency departments (ED) in China are often available to patients with non-severe, non-urgent conditions on a first-come-first-served basis [31]. Within a complex healthcare system, the impact of ASPs on clinical antibiotic use remains unknown.

This study aimed to evaluate the impact of the above ASPs with a focus on the policy’s targeted IV antibiotic use levels in secondary and tertiary hospitals in Zhejiang province. We conducted a mixed-methods study including individual interviews with three types of key stakeholders – healthcare workers, patients, and policymakers in two cities – as well as a case study using a combined questionnaire survey and routine hospital-based data collection.

## Methods

This study was conducted in four hospitals: two from an economically developed city A and the other two from an economically developing city B in Zhejiang Province. The mixed-methods study consisted of two phases: a qualitative study (Phase 1) using individual interviews with health staff and patients from the four hospitals, and policymakers from the two cities and a case study (Phase 2) in one of the participating hospitals collecting survey data and hospital routine data.

### The qualitative study (phase 1)

We purposively selected two policymakers/implementers from local health bureaus in each city. Two hospitals were purposively selected in each city, and ten doctors and two nurses from OD and ED, resulting in twenty doctors and four nurses from OD and ED respectively, in each hospital were invited to participate in an individual interview. With the assistance from the emergency doctors and nurses, we identified and selected four patients in each hospital who were referred to the ED by doctors in other ODs or self-referred to the ED to get an IV antibiotic. A total of four policymakers, forty doctors, eight nurses, and sixteen patients were interviewed. Topic guides with a focus on IV antibiotic use were developed to facilitate interviews. For policymakers, we focused on the achievements, the problems, and possible solutions surrounding the implementation of the ban. For doctors and nurses, the topic guide included their views about the ban, changes of doctors’ IV antibiotic prescribing, patients’ reactions towards the ban, and subsequent changes of their health service seeking behaviours. For patients, the topic guide included their knowledge, views, experiences of referral to ED for IV antibiotics use, and satisfaction with the ban. The detailed interview guide is available in Additional file 1.

The sampled cities and hospitals were assured to be de-identified in any reports. Policymakers/implementers were interviewed at their offices for 30 to 50 min. The face-to-face interviews with doctors, nurses, and patients lasted for 40 to 60 min and were conducted in private hospital meeting rooms. All participants were informed of the purpose and content of the study. Participation in the interview was voluntary and they could withdraw any time during the study without being affected in any ways. The confidentiality was stressed before the commencement of the interviews. All participants gave written consent and interviews were recorded digitally.

Recordings were transcribed verbatim. Data were coded by two research assistants and analysed by two authors. Thematic framework analysis was conducted following the method proposed by Braun and Clarke [32]. The two authors coded independently and met regularly to discuss candidate themes. Disagreements were solved through group meetings with other investigators in the team. The final set of themes was reviewed by another researcher familiar with the transcripts to check that they accurately reflected the content. Key themes that emerged from the transcripts were then incorporated into the questionnaire for the survey with doctors in a selected hospital in Phase 2.

### The quantitative case study (phase 2)

With consent from one of the sampled hospitals, we collected the hospital’s routine data and invited all the doctors who have been working in the OD, the ED or both since the ban was initiated. Using the hospital information system, we extracted routine data one year before (May 2015 to April 2016) and one year after (May 2016 to April 2017) the ban started. The hospital routine data included the number of patients visits, the total number of outpatient antibiotics prescriptions, oral and IV antibiotic prescriptions, and combination antibiotic therapy in OD and ED.

The questionnaire was pre-tested with 20 doctors through face-to-face interviews. Minor amendments to improve readability and layout were made according to their feedback. All eligible doctors in ODs and EDs were invited to participate in the survey. The survey content focused on the impact of the ban and doctors’ responses to patients’ demand for IV antibiotic use.

The quantitative data were analysed using IBM SPSS V20. Descriptive analyses were conducted. Chi-square tests were used to measure the changes in antibiotic prescribing before and after the ban. We did not conduct imputation because the extent of missingness was minimal (e.g., below 1%).

## Results

### Socio-demographic characteristics of respondents (Table 1)

For the qualitative interview (Phase 1), all four policymakers/implementers had a bachelor’s degree, with a mean age of 40.6 years (SD = 10.3). Of the 48 health workers (mean age = 36 years, SD = 6.7), the majority were male (60.4%) and had a bachelor’s degree (83.3%). The mean age of 16 patients interviewed was 49.9 (SD = 14.3) years, and more than half of them were males.

**Table 1:** Socio-demographic profile of the respondents

	Qualitative study (Phase 1) (*n* = 68)			Case study (Phase 2) (*N* = 207)
	Policymakers *n* = 4	Health workers *N* = 48	Patients *N* = 16	
Gender				
Male	3 (75.0)	29 (60.4)	9 (56.3)	125 (60.4)
Female	1 (25.0)	19 (39.6)	7 (43.7)	82 (39.6)
Age				
Mean (SD)	40.6 (10.3)	36.0 (6.7)	49.9 (14.3)	39.8 (8.5)
Education level				
Junior College	0	5 (10.4)		
Bachelor’s degree	4 (100.0)	40 (83.3)		179 (86.5)
Master’s degree and above	0	3 (6.3)		28 (13.5)
Participants				
Outpatient doctor		20 (41.7)		
Emergency doctor		20 (41.7)		
Nurse		8 (16.7)		
Working experiences before the ban implementation				
Both OD and ED				84 (40.6)
Only OD				94 (45.4)
Only ED				29 (14.0)

For the survey (survey data, Phase 2), we invited all 340 eligible doctors, and 245 questionnaires were returned (response rate = 72.1%). Among the 245 questionnaires, 38 (15.5%) were discarded because of inconsistencies in the logical questions. Among the 207 remained respondents, the mean age was 39.8 (SD = 8.5) years. Most of them were male (60.4%) and had a bachelor’s degree (86.5%). Ninety-four (45.4%) and 29 (14.0%) worked in the OD or the ED only, respectively, and the rest 84(40.6%) worked in both the OD and the ED since May 2016.

### The change of antibiotic prescribing in ODs

#### Qualitative data (phase 1)

Most of the doctors and nurses in ODs thought that the number of patients who used IV antibiotics reduced substantially after the ban.

*I perceived a significant reduction in the number of IV antibiotic prescription in the OD since we are not allowed to prescribe IV antibiotics for adult patients in the OD.* (Outpatient doctor)

*Before the ban, the OD infusion room was the most crowded place in the hospital. The effect of the ban is obvious, and our workload indeed reduced.* (Nurse in OD)

#### Case study (hospital routine data, phase 2)

The routine data from the hospital (Table 2) also showed that the total antibiotic use rate in the OD decreased from 19.9% (145 thousand out of 728 thousand prescriptions) to 17.8% (128 thousand out of 721 thousand prescriptions) (*p* < 0.001). The total IV antibiotic use rate in the OD reduced from 8.6% (62 thousand out of 728 thousand) to 4.7% (34 thousand out of 721 thousand) (p < 0.001), but the oral antibiotic use rate increased from 11.6% (84 thousand out of 728 thousand prescriptions) to 13.2% (95 thousand out of 721 thousand prescriptions) (*p* < 0.001). Despite the reduction in IV antibiotic use rate in the OD, there were still 34 thousand IV antibiotic prescriptions in paediatrics, which accounts for 35.4% of IV antibiotic prescriptions in both OD and ED after the ban. The ban not only reduced the IV and total antibiotic use but also decreased the amount of restricted- and special-grades antibiotics and combination antibiotic therapy. The restricted- and special-grade antibiotic use rate decreased from 39.8% (57 thousand prescriptions) to 32.5% (41 thousand prescriptions) and 2.4% (3 thousand prescriptions) to 0.1% (100 prescriptions) (*p* < 0.001), respectively. The rate of combination antibiotic therapy reduced from 17.7% (25 thousand prescriptions) to 11.3% (14 thousand prescriptions) (*p* < 0.001).

**Table 2:** Changes of antibiotic prescribing behaviors in the case study hospital (thousand)

	Before (*n* = 728)	After (*n* = 721)	χ^2^	*p*-value
Total antibiotic prescription (rate^a^)	145 (19.9)	128 (17.8)	1015	< 0.001
IV antibiotic prescription (rate^b^)	62 (8.6)	34 (4.7)	8473	< 0.001
Oral antibiotic prescription (rate^c^)	84 (11.6)	95 (13.2)	844.3	< 0.001
	(*n* = 145)	(*n* = 128)		
Classified antibiotic use rate ^d^				
Non-restricted	94 (65.4)	90 (70.1)	706.1	< 0.001
Restricted	57 (39.8)	41 (32.5)	1558	< 0.001
Special	3 (2.4)	0.1 (0.1)	2655	< 0.001
Antibiotic combination therapy ^e^	25 (17.7)	14 (11.3)	2231	< 0.001

^a^Total antibiotic prescription rate = the number of antibiotic prescriptions/total number of prescriptions;

^b^IV antibiotic prescription rate = the number of IV antibiotic prescriptions/total number of prescriptions;

^c^Oral antibiotic prescription rate = the number of oral antibiotic prescriptions/total number of prescriptions;

^d^Classified antibiotic use rate = the number of classified-grade antibiotic prescriptions/total number of antibiotic prescriptions;

^e^Antibiotic combination therapy = the number of prescriptions with two or more antibiotic/total number of antibiotic prescriptions

### Policy implementation: the balloon metaphor [33]

#### Qualitative data (phase 1)

**Fig. 1:**
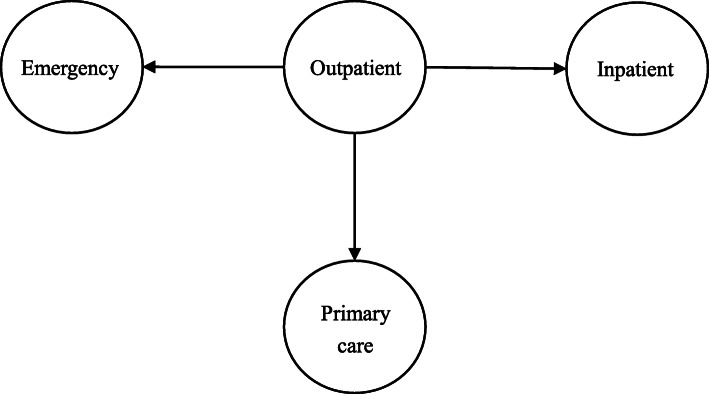
Patient flow after the ban in OD

When the doctors were not allowed to prescribe the IV antibiotic for the adult patients in the OD, they referred the patients who clinically needed or demanded IV antibiotics to the ED, a primary care facility, or an inpatient ward (Fig. 1). Twelve out of 19 ED doctors thought that the number of ED visits increased sharply after the launch of the ban. Some patients went directly to the ED after they became aware that the IV antibiotic use was not allowed in the OD.

*Since the ban was implemented, the outpatient doctors in our hospital referred their patients who expected IV antibiotics to the ED. It did increase our workload. I had ten to twenty more patients who were referred from the OD every day after the ban was launched.* (Emergency doctor)

*It happened to my son once. The OD doctor told him that he needed to use IV antibiotics, but they were not allowed to prescribe it. So, he had to go to the ED again for prescribing IV antibiotics.* (Patient)

Some doctors disclosed that some outpatients who needed IV antibiotic were referred to the inpatient ward.

*I work in the Hospitalization Preparation Center (which helps coordinate hospital resources for admitting patients). The ban increased the number of patients referred from the OD to the inpatient ward. For example, after the implementation of the ban, there were at least four to five patients every day, who might not clinically need it, were hospitalized to get IV antibiotics.* (Outpatient doctor)

Some flowed to the primary care facilities for a prescription of IV antibiotics.

*The patients were not happy when I told them they could not get the IV antibiotics in the OD. They left the hospital and got it in the community health center. The community health center has fewer patients and does not have a ban on IV antibiotic use.* (Outpatient doctors)

#### Case study (survey data, phase 2)

Survey findings from the case study triangulated the qualitative interview findings. Table 3 showed that among the 178 doctors who have worked in the OD after the ban implementation, the overwhelming majority had encountered patients who did not need IV antibiotic prescriptions but demanded one in the OD. Only one-third of the study participants reported that they never referred patients to the ED for IV antibiotics, whereas the remaining two-thirds indicated “often”, “sometimes”, and “occasionally”. Similarly, more than half of doctors reported that they had referred the patients to primary health care facilities; and another half indicated they had hospitalized the patients. Notably, nearly all the 113 doctors who worked in the ED after the ban implementation reported that they had seen patients who were referred from the OD to get an IV antibiotics prescription, and 96.5% of doctors reported they had seen patients who visited ED directly only for IV antibiotics when they knew they could not get IV antibiotics in the OD.

**Table 3:** Flow of patients in the OD after the ban

**Questions for the OD doctors (*n* = 178)**	Often n(%)	Sometimes n(%)	Occasionally n(%)	Never n(%)
After the ban implementation, how often did you see patients who did not need IV antibiotics but demanded one in the OD from you?	37 (20.8)	75 (42.1)	59 (33.1)	7 (3.9)
After the ban implementation, how often did you see patients who demanded IV antibiotics in the OD from you and you referred him or her to the ED in the hospital?	23 (12.9)	71 (39.9)	55 (30.9)	29 (16.3)
After the ban implementation, how often did you see patients who demanded IV antibiotics in the OD from and you referred him or her to the primary care?	14 (7.9)	48 (27.0)	46 (25.8)	70 (39.3)
After the ban implementation, how often did you see patients who demanded IV antibiotics in the OD from you and you referred him or her to an inpatient ward in the hospital?	1 (0.6)	41 (23.0)	44 (24.7)	92 (51.7)
**Questions for the ED doctors (*n* = 113)**	Often	Sometimes	Occasionally	Never
After the ban implementation, how often did you see patients who were referred by other doctors from OD to ED only to get an IV antibiotics prescription?	44 (38.9)	47 (41.6)	20 (17.7)	2 (1.8)
After the ban implementation, how often did you see patients who visited ED for the purpose of getting an IV antibiotics prescription?	47 (41.6)	46 (40.7)	16 (14.2)	4 (3.5)

### Impact on emergency services delivery

The doctors in our study thought that patient flow into the ED for IV antibiotics had made the triage functioning in ED worse. The increased number of patients with simple conditions in the ED abused hospital services, causing wastage and inefficiency.

*The ED is for patients with emergency and severe conditions. However, patients crowded into ED to get IV antibiotics after the ban. Especially during flu seasons, a large number of OD patients came to the ED which abused the limited resources for patients who did need emergency care.* (Emergency doctor)

*Some patient with a common cold would come to ED to get IV antibiotics, and they won’t take our advice that they do not need one. Many patients had terrible attitudes and would argue with us. I have to give up persuading and assigned him/her to a doctor.* (Triage nurse)

*Sometimes patients in the OD did need to use IV antibiotics according to my judgment, and sometimes patients asked for using IV antibiotics, but I failed to convince them to give up. I had to refer them to the ED for IV antibiotic prescriptions. It sometimes happened that the ED doctors were unconfident to treat my referred patients because they were not trained in my specialization. The ED doctors then called me and worked with me to treat the patients. It wasted our time and the hospital’s resources.* (Outpatient doctor)

### The barriers to the implementation of the ban

#### The radical policy implementation

Although the ban aimed to reduce the IV antibiotic overuse in Chinese health facilities, most doctors thought that banning IV antibiotic use in the OD completely without a buffering period was unreasonable. However, the policymakers thought the radical approach was necessary to rationalize the IV antibiotic use in the OD.

*I don’t agree with the ban implementation approach. It would be better to give the patients some time to understand and accept the ban.* (Outpatient doctor)

*If we don’t stop IV antibiotics use completely in the OD and once open up an exemption, then every doctor wants an exception. Consequently, we might not be able to reduce IV antibiotic use as we expected. It is the only realistic and practical way to decrease the IV antibiotic use rate by completely banning IV antibiotic use [in ODs].* (Policymaker)

#### Doctors circumvented the regulations

Doctors complained that the policy’s requirements of performance targets of antibiotic prescriptions had seriously harmed clinical autonomy in their daily practices.

*When I prescribe medicines for patients, I have to not only worry about whether the illness will be cured and how long it will take, but also worry about the percentage of medicine cost out of the total cost per prescription, the antibiotic use rate, and consider how much revenues I make for my hospital. I feel like I need to be not only a doctor but also an actuary.* (Outpatient doctor)

To reach the target percentages set by the Health Bureau, hospitals supervised every individual doctor’s antibiotic prescribing rate every month. Consequently, there was a financial penalty if s/he did not achieve the target percentage. Some doctors found countermeasures to meet those target indicators and avoid the financial penalties rather than de-facto improvement in prudent antibiotic use.

*Our hospital calculates the antibiotic use rate for each doctor every month. If anyone exceeds the rate, s/he will be fined. At the end of each month, I sometimes had to call my relatives and friends to visit me and prescribed them medicines without antibiotics. Therefore, my antibiotic prescribing rate was reduced because the prescriptions for my relatives and friends increased the denominator.* (Outpatient doctor)

*When I met some patients asking for antibiotic use, I sometimes prescribed the patient with more diagnostic tests when I run out of my quota to prescribe antibiotics. After the patient completed the tests and got the results, it had been in the afternoon or the next day when I have been off work. So it delayed prescribing antibiotics and transfer it to other doctors. [If the patient were prescribed with antibiotics] it did not count on my head.* (Outpatient doctor).

*At the beginning days of a month, most of the patients who expected antibiotics from me would get one. However, for those patients who come in the remaining days of a month when the hospital calculates our antibiotic prescription rate, it is difficult for me to prescribe them because I would exceed my quota in that month. I would refer them to other doctors who still have some quota left if the patient does need antibiotics.* (Outpatient doctor)

#### Lack of doctor-patient communication during encounters

When the patients asked for IV antibiotics in the OD, most doctors responded to those patients by referring them to other services directly where IV antibiotics were available rather than properly educating the patient. There were two main reasons. Firstly, it was more time-consuming to conduct health education.

*For most of the outpatient doctors, our consultation workloads have already been heavy. We would rather spend two minutes to refer the patients who insist on using IV antibiotics to the ED than spending another 10 to 15 minutes to educate and convince the patient not to use IV antibiotics.* (Outpatient doctor)

*I do not have much time to communicate with patients. If patients come to demand IV antibiotics, I will prescribe one.* (Emergency doctor)

Secondly, poor doctor-patient relationship in China was believed to be an obstacle to effective doctor-patient communication. Due to the perceived poor doctor-patient relationship, doctors were inclined to meet patient expectations and became reluctant to address any potential patient complaints.

*I need to be very careful when I explained the policy of IV antibiotic use ban and tried to educate the patients not to use unnecessary IV antibiotics. Sometimes patients thought I was the person who did not allow them to use IV antibiotics. If anything unexpected happens to the patients, they think that I must take the responsibility.* (Emergency doctor)

*If I refuse to prescribe some patients with IV antibiotics, they will say: 'If you don’t prescribe infusion for me and if anything happened to me, you need to take the responsibility.’ The disease itself is complicated, you know. There are many situations out of my control. I can’t take the risk.* (Emergency doctor)

## Discussion

Tackling IV antibiotics overprescribing is a top priority of China’s ASPs initiative. One of the key measures adopted is a top-down health policy that completely bans IV antibiotic use in outpatient departments, starting with tertiary hospitals. To the best of our knowledge, our study was the first to evaluate the influence of such a policy in Zhejiang Province. We found that the ban has successfully reduced the gross antibiotic prescriptions, IV antibiotic use, and combination antimicrobial therapy in ODs. Fewer restricted and special grades antibiotics were prescribed as well. However, unintended effects were observed. These included driving some patients to health services which had less strict control of IV antibiotic use, such as ED, inpatient services, and primary care facilities, subsequently causing more wastage of health resources at these service sections.

Firstly, we found a positive impact of the ban on the reduction of the total antibiotic use, IV antibiotic use, and combination antimicrobial therapy. This is consistent with Gong et al. [34] that the top-down antimicrobial stewardship regulations appeared to be effective in reducing the gross antibiotic overuse. Both qualitative interviews and hospital routine data revealed that IV antibiotic use, administration of advanced antibiotics, and combination antibiotic therapy were significantly reduced after the ban implementation. However, given the ways that doctors managed to get around the policy and the number of prescriptions been manipulated remained unknown, future quantitative study is needed to support the finding that the number of IV antibiotics decreased. Additionally, there was a significant rise in oral antibiotic use. Such a risk compensation is expected. The mode of oral antibiotic use is associated with a lower risk for AMR and presents fewer harms to patients relative to IV antibiotics [35, 36]. However, both the mode of IV antibiotics administration and the overuse or misuse of oral antibiotics fuel the AMR crisis. Moreover, at present the ban only focused on adult patients and did not target paediatric patients, antibiotic overuse among child patients remains a huge challenge. Our data showed that paediatric antibiotic prescriptions accounted for a large proportion of the total antibiotic use in both OD and ED. Studies found that children who received unnecessary antibiotic therapy were more likely to develop chronic disease, including obesity, asthma, juvenile idiopathic arthritis, and celiac disease, in the future [37]. Misuse of antibiotics in paediatric populations is also a significant public health issue and merits equal if not more attention. Additionally, actions to mitigate the risk compensation of oral antibiotic misuse are needed.

Secondly, we also found unintended consequences among which patient flow to other service departments or sectors, either through self-referral or a physician referral, for IV antibiotics became the most prominent. A similar balloon metaphor phenomenon was reported in medical cost control and antibiotic resistance before [33], meaning constraining one end causes the other end to bulge. The balloon metaphor is common during the Chinese health policy implementation processes. For example, China introduced its national Essential Drug List and implemented a zero-mark-up policy in 2009 to curb drug abuse and promote the availability, safety, and rational use of essential drugs [38] which led to reduced drug expenditures but more diagnostic tests with no measurable changes in total health expenditures [39]. Likewise, the complete ban of IV antibiotic use among adult patients in ODs unexpectedly caused services abuse in ED and inpatient sections. Due to the malfunctioning triaging services in EDs in China’s hospitals, medical conditions are not adequately evaluated based on the severity or urgency for most patients [31, 40]. Many patients with common simple conditions can also see a physician and get treated in an ED on a first-come-first-served basis. Hence, referring patients to the ED simply for IV antibiotics led to abuse of emergency services which should prioritize those with urgent, severe, or life-threatening conditions. Further, some physicians might feel that IV antibiotic was necessary for a patient and admit the patient into an inpatient ward to administer the treatment. Primary care facilities were another direction where patients flow to for IV antibiotics. The necessity of these referrals, however, remained unknown owing to the lack of quality assessment of these encounters. Future efforts are needed to reduce unnecessary referrals and better coordinate for patients who clinically need IV antibiotics.

Thirdly, complaints and concerns about the radical policy implementation without a buffering period were common among physicians in ODs. Besides, physicians had their way to circumvent the policy. Without prescribing quality assessment, these countermeasures adopted by physicians caused concerns about the reliability of such digital indicators as a main quality control measure. It is worth policymakers’ revisit of the reliability of these digital indicators and consideration of integrating quality assessment of randomly selected prescriptions to improve the clinical rationality of antibiotic prescribing. More importantly, AMR is a public health risk that requires multisectoral efforts to tackle, including health care providers and service users as the two key stakeholders. However, the current policy led to an unexpected shift in the “location” of risk (i.e., the risk of AMR to the risk of doctors’ professional standing). Doctors faced risks of a fine following unmet hospital performance evaluation criteria, fear of violence from dissatisfied patients, and constrained time for patient communication in the clinical settings. These factors drove doctors to simply adopt the above risk-shifting behaviours which may compromise their professionalism. We found that within the hospitals, the risk of AMR shifted from one doctor to another doctor in OD or from the OD to the ED, primary care, and inpatient services.

Lastly, the policy focused on the supply side only and neglected the patient side. Previous research showed that Chinese patients held high expectations on IV antibiotics [41, 42], suggesting the importance of properly managing patients’ unreasonably high expectations in reducing IV antibiotics without generating patient dissatisfaction. Therefore, supportive measures targeting the demand side are needed when shaping the ASP programme or policy. Nevertheless, public health education campaigns to improve patients’ awareness of AMR and the importance of rational antibiotic use are lacking. Managing patients’ demand for IV antibiotics is worth more efforts. Doctor-patient communication on IV antibiotic use is also inadequate due to limited consultation time for each patient. Physicians’ perceived intense doctor-patient relationship in China’s health system, characterized by a high prevalence of patient violence against doctors [43], is a contributor to physicians’ defensive medicine, reluctancy to confront patient demand for IV treatment, and their subsequent prescriptions to meet patients’ expectations [44]. Potential directions may be addressing behavioural and social influences which are often ignored in ASPs [44–46]. Specifically, the perverse incentives for doctors to overprescribe in the Chinese setting [8], low self-efficacy in antibiotic prescribing practices among physicians [47, 48], poor doctor-patient communication skills, poor antibiotic-related knowledge and high expectations among patients [42], and the intense doctor-patient relationship [49] could be good starting points.

All in all, despite some positive effects, banning IV in outpatient services at hospitals is not a panacea, and general public education to improve public understanding is also essential for minimizing unintended adverse consequences such as patient flow to other service departments or sectors for unnecessary antibiotic treatment. A socio-ecological model to address the determinants for IV antibiotic use from the individual, community, institutional, and policy levels along with a communication for development approach [50] for intervention or policy design targeting determinants from different levels are recommended. Policymakers and researchers need to keep the entire ecology of the AMR crisis in mind that the public health goal is not only to reduce IV antibiotic use but also minimize the misuse of oral antibiotics.

This study has some limitations. First, we cannot identify narrow-spectrum and broad-spectrum antibiotics in the health information system to assess the appropriateness of antibiotic therapy. However, we used the non-restricted, restricted, and special antibiotics classified by the Chinese NHC which to some extent illustrated the improvement in antibiotics use. Secondly, the hospital records we obtained did not include lab results about the bacteria or other pathogen a patient had (if any) to enable us to assess the appropriateness of antibiotic therapy. Thirdly, the survey with the doctors about whether they would refer patients to other departments to get IV antibiotics was a sensitive topic for them. Thus, respondents might give socially desirable answers in a self-administrated questionnaire. However, the reported percentages of referring patients to other departments were high, which suggested that they have been quite honest, and the survey should be a success. Nevertheless, we conducted a qualitative study, and the results were consistent with the survey findings. Lastly, our study only targeted the effect of the ban in Zhejiang province, an economically developed region, which may not be representative of poorer areas in China. However, we selected multiple sites, facilities, and enrolled different stakeholders in our study. The findings may be useful to inform future research about other areas of the nation or countries with similar settings.

## Conclusions

Our study has several implications for China’s national ASPs. A complete ban of IV antibiotic use in OD may successfully lower gross antibiotic use, IV antibiotic use, and combination antibiotic therapy, but increased oral antibiotic use in adult and neglected paediatric antibiotic overuse represents challenging unintended consequences. Further, more research attention to other unintended consequences is highly warranted. These unexpected consequences included more patient flow to ED, inpatient ward, and primary care services with the main purpose of obtaining IV treatment, along with physicians’ risk-shifting behaviours. Multi-faceted interventions targeting both health care providers and service users were highly recommended to facilitate behavioural change and improve rational antibiotics use among the two key groups [51]. Future efforts may include 1) to de-escalate both oral and IV antibiotic therapy in paediatric and reduce oral antibiotic therapy among adults in outpatient clinics, 2) to reduce unnecessary referrals by OD doctors to ED, primary care, or inpatient services and better coordinate for patients who clinically need IV antibiotics, 3) to incorporate demand-side tailored measures, such as public education campaigns, and 4) to improve doctor-patient communication. Future research is needed to be done to understand how primary care and other community clinics implement the ban.

## Supplementary Information

**Additional file 1 Interview guide**. File providing the details of the questions designed and used as a study tool during the interviews.

## Data Availability

The datasets generated during and/or analyzed during the current study are not publicly available but are available from the corresponding author upon reasonable request.

## Abbreviations

AMR: Antimicrobial Resistance
IV: Intravenous
OD: Outpatient Department
ED: Emergency Department
ASPs: Antimicrobial Stewardship Programs
NHC: National Health Commission
