# Supplementary material for: The influence of a ban on outpatient intravenous antibiotic therapy among the secondary and tertiary hospitals in China
Source: BMC Public Health. 2020 Nov 25;20:1794. doi: 10.1186/s12889-020-09948-z (PMC7690204; doi:10.1186/s12889-020-09948-z)
Supplement: Supplementary file 1 — Additional file 1 Interview guide. File providing the details of the questions designed and used as a study tool during the interviews. [file 12889_2020_9948_MOESM1_ESM.docx]

**Interview guide for health workers (doctors and nurses)**

**The socio-demographics**

*Would you mind starting by telling me a bit about yourself?*

1. What is your age?
2. What is your education attainment (the highest level of education completed)?
3. How many years have you been working in this hospital?
4. Which department are you working in?
5. Please tell me something about your previous working experiences. How many departments (in this hospital or other hospitals) have you worked in so far?

**Questions related to IV antibiotic use ban**

*Please tell me what you currently know about the outpatient* (OD) *IV antibiotic use ban.*

1. How do you think about the ban on outpatient IV antibiotic use? What are the pros and cons of the ban?

2. Have you ever encountered any problems after the implementation of the ban in your hospital?

3. What did you do if you find an OD patient who needs an IV antibiotic? (OD doctor)

4. Were there any disease that did need IV antibiotic use during your consultations?

5. What did you do if patients requested IV antibiotic use after the implementation of the ban? (OD doctor)

6. What is the biggest challenge for your work after the implementation of the ban?

7. Have you discussed it with your colleague about the ban? If yes, how did they think? Were they satisfied with the ban?

8. How did the leadership of your hospital think of the ban?

9. What will happen if you fail to persuade an OD patient to give up using IV antibiotics?

10. Please describe the patients’ reaction when you told them IV antibiotic is not allowed to use in the OD.

11. Have you offered health education for patients who requested IV antibiotics? How about your colleagues in your department? If not, please tell me the barriers to educate patients during the consultation.

12. Were there any changes in health service-seeking behaviours among patients after the ban?

13. Do you have any suggestions to improve the ban policy or to reduce IV antibiotic use?

**Interview guide for patients**

With the assistance from the emergency doctors and nurses, we identified and selected four patients in each hospital who were referred to the Emergency Department (ED) by doctors in other ODs or self-referred to the ED to get IV antibiotics.

**The socio-demographics**

*Would you mind starting by telling me a bit about yourself?*

1. What is your age?
2. What is your education attainment (the highest level of education completed)?

**Questions related to IV antibiotic use ban**

1. What kind of health facilities did you visit the most (primary, secondary, tertiary), and why?

2. Have you heard of the word “antibiotics”? If yes, please tell me more about antibiotics.

3. Do you know IV antibiotic use in the OD is not allowed in this hospital? If yes, where or from whom did you get the information?

4. How do you think of the ban?

5. Please tell us your experience of referral from OD to ED for IV antibiotics?

6. Were you satisfied with the ban? Why?

7. Did the OD doctor advise you not to use IV antibiotics?

**Interview guide for policymakers/implementers**

**The socio-demographics**

*Would you mind starting by telling me a bit about yourself?*

1. What is your age?
2. What is your education attainment (the highest level of education completed)?
3. How many years have you been working in the Bureau of Health?

**Questions related to IV antibiotic use ban**

*Could you please tell me something about the ban on IV antibiotics in your city?*

1. When was the ban implemented in your city?

2. How many and what kind of health facilities were covered by the ban?

3. What do you think of the ban in general?

4. Please describe the operation of the policy implementation in your city.

5. What were the most common feedback and response from the hospitals after implementing the ban?

6. Please tell me the achievements and challenges of the ban.

7. Do you have any suggestions to improve the ban or to reduce IV antibiotic use?
